# Robust Humoral and Cellular Immune Responses to Pertussis in Adults After a First Acellular Booster Vaccination

**DOI:** 10.3389/fimmu.2018.00681

**Published:** 2018-04-04

**Authors:** Saskia van der Lee, Debbie M. van Rooijen, Mary-Lène de Zeeuw-Brouwer, Marjan J. M. Bogaard, Pieter G. M. van Gageldonk, Axel Bonacic Marinovic, Elisabeth A. M. Sanders, Guy A. M. Berbers, Anne-Marie Buisman

**Affiliations:** ^1^Centre for Infectious Disease Control, National Institute for Public Health and the Environment (RIVM), Bilthoven, Netherlands; ^2^Department of Paediatric Immunology and Infectious Diseases, Wilhelmina Children’s Hospital, University Medical Centre, Utrecht, Netherlands

**Keywords:** pertussis, adult immunization, antibody decay, memory B-cells, memory T-cells

## Abstract

**Introduction:**

To reduce the pertussis disease burden, nowadays several countries recommend acellular pertussis (aP) booster vaccinations for adults. We aimed to evaluate the immunogenicity of a first adult aP booster vaccination at childbearing age.

**Methods:**

In 2014, healthy adults aged 25–29 years (*n* = 105), vaccinated during infancy with four doses of whole-cell pertussis (wP) vaccine, received a Tdap (tetanus, diphtheria, and aP) booster vaccination. Blood samples were collected longitudinally pre-booster, 2 and 4 weeks, and 1 year and 2 years post-booster. Tdap vaccine antigen-specific antibody levels and memory B- and T-cell responses were determined at all time points. Antibody persistence was calculated using a bi-exponential decay model.

**Results:**

Upon booster vaccination, the IgG levels specific to all Tdap vaccine antigens were significantly increased. After an initial rapid decline in the first year, PT-IgG antibody decay was limited (15%) in the second year post-booster. The duration of a median level of PT-IgG ≥20 IU/mL was estimated to be approximately 9 years. Vaccine antigen-specific memory B- and T-cell numbers increased and remained at high levels although a significant decline was observed after 4 weeks post-booster. However, Th1, Th2, and Th17 cytokine production remained above pre-booster levels for 2 years.

**Conclusion:**

The Tdap booster vaccination in wP-primed Dutch adults induced robust long-term humoral and cellular immune responses to pertussis antigens. Furthermore, PT-IgG levels are predicted to remain above the presumed protective cut-off for at least 9 years which might deserves further attention in evaluating the current recommendation to revaccinate women during every new pregnancy.

## Introduction

The incidence of clinical pertussis cases strongly declined after the introduction of whole-cell pertussis (wP) vaccines in infant national immunization programs (NIP) in the 1940 and 1950s (1). Despite a consistently high vaccination coverage, an increase in numbers of pertussis cases is observed in many countries and in all age groups (2–5). Consequently, in addition to pre-school acellular pertussis (aP) booster vaccinations, several countries have implemented aP booster vaccinations for adolescents and adults (6–8). Furthermore, to protect unvaccinated infants against pertussis, pregnant woman are advised to receive maternal aP booster vaccination in more than 25 countries (9–11).

Waning immunity after vaccination and natural infection as well as the switch from wP to aP vaccines in the primary infant vaccination series are thought to have contributed to the pertussis resurgence (12–14). The switch from wP to aP vaccines for infants occurred in January 2005 in the Netherlands, which is rather late compared with other high-income countries. After an aP vaccination in adults, low pertussis antibody levels, and in particular low anti-pertussis toxin (PT) antibodies, that are considered the most protective against clinical *B. pertussis* symptoms, have been observed already within 1 year (15, 16). These studies were, however, conducted in a period with a presumed lower circulation of *B. pertussis* (late 1990s) in comparison to the past decades (17, 18).

Nowadays, increased circulation of *B. pertussis* might allow for more natural boosting of the immune system, and affect antibody kinetics as well as cellular immunity after an adult aP booster vaccination. We aimed to evaluate the long-term immunogenicity of a first adult aP booster vaccination at childbearing age. A bi-exponential antibody decay model was used to predict the duration of antibody persistence to PT after vaccination. This study provides valuable information for the improvement of adult and maternal pertussis vaccination programs.

## Materials and Methods

### Study Design and Participants

In this phase IV, longitudinal intervention study, healthy Dutch adults 25–29 years of age were recruited to receive a tetanus, diphtheria, and acellular pertussis (Tdap) booster vaccination. Exclusion criteria were pregnancy at the start of the study; present severe disease or medical treatment that might interfere with study results; an adverse event after previous vaccinations; other pertussis vaccinations than those given according to the Dutch NIP; diphtheria and/or tetanus vaccination in the past 5 years; plasma products received in the past 6 months; any vaccination in the last month and/or antibiotic use or fever (≥38°C) in the 2 weeks before study enrollment. Written informed consent was obtained at the start of the study. The study was approved by the Medical Ethics Review Committee North Holland (METC-NH, Alkmaar, the Netherlands) and registered at the European clinical trials database (2013-005355-32) and the Dutch trial register (www.trialregister.nl; NTR4494).

### Vaccination Background

All participants had received the Dutch diphtheria, tetanus, whole-cell pertussis, and inactivated poliovirus combination vaccine (National institute for Public Health, Bilthoven, the Netherlands) according to the then NIP at 3, 4, 5, and 11 months of age. In this study, the participants received a Tdap booster vaccine (Boostrix™, GlaxoSmithKline, Rixensart, Belgium). The vaccine contained 8 µg PT and filamentous hemagglutinin (FHA), 2.5 µg pertactin (Prn), ≥2 IU diphtheria toxoid (Dd), and ≥20 IU tetanus toxoid (Td).

### Blood Samples

Serum samples were collected just before, 14 days (±2 days), 28 days (±2 days), 1 year (±2 weeks), and 2 years (±2 weeks) after the Tdap booster vaccination. Sera were stored at −20°C until analysis. From a randomly selected subset of 60 participants, additional blood was sampled in vacutainer cell preparation tubes containing sodium citrate (Becton Dickinson (BD) Biosciences, San Jose, CA, USA). PBMCs were isolated within 16 h, and stored at −135°C as described previously (19).

### Serological Analysis

PT-, FHA-, and Prn-specific IgG and IgA, and Dd- and tetanus toxin (TT)-specific IgG antibody concentrations were quantified using the fluorescent-bead-based multiplex immunoassay (MIA) as described (20–22). To express pertussis-IgG and IgA concentrations in international units (IU) per mL, the WHO international standard (pertussis antiserum first international standard, 06/140, NIBSC) was used. A PT-IgG concentration of 20 IU/mL was used as an arbitrary cut-off for protection (23) and 50 IU/mL to indicate an infection with pertussis in the preceding years (17, 20). An IgA concentration ≥1 IU/mL was used as seropositive.

From 42 longitudinal samples, the PT- and Prn-IgG avidity was determined using the MIA with minor modifications (24), using 1.5 M (for PT) and 2.5 M (for Prn) ammonium thiocyanate (NH_4_SCN). The geometric mean avidity index (GMAI) was expressed as the percentage of antibodies that remained bound to PT- or Prn-conjugated beads after NH_4_SCN treatment in comparison to untreated (PBS) samples.

### Flow Cytometry

The absolute numbers of circulating B-cells and B-cell subsets were determined in 60 paired samples before and 2 weeks after the booster vaccination with a lyse-no-wash protocol using TruCOUNT tubes (BD Biosciences). The fluochrome-conjugated antibodies CD19(J3-119)-PE-Cy7 (Beckman Coulter, Fullerton, CA, USA), CD27(M-T271)-BV421, IgD(IA6-2)-FITC (both from Biolegend, San Diego, CA, USA), and CD38(HB7)-APC-H7 (BD Biosciences) were used. Samples were measured using a LSRFortessa flow cytometer (BD Biosciences). The B-cell population in PBMCs before and after culture was determined using CD19-PerCPCy5.5 (BD Biosciences), and samples were measured on a FacsCanto flow cytometer (BD Biosciences). Data were analyzed using FACSDiva™ v8 (BD Biosciences) and FlowJo v10 (FlowJo company, Ashland, OR, USA) with a gating strategy as described (25).

### Antigen-Specific B- and T-cell Responses

From 30 participants, vaccine antigen-specific B- and T-cell responses were determined. For B-cell responses, PBMCs were polyclonally stimulated for 5 days after which the number of specific IgG memory B-cells/10^5^ CD19^+^ cells was determined in PT-, FHA-, Prn-, and Td-specific ELISpot assays (19). Per participant, samples of different time points were determined simultaneously. Lower limit of quantification was 0.5 spots/10^5^ CD19^+^ cells.

For T-cell responses, PBMCs were stimulated for 5 days with PT (heat inactivated), FHA, Prn, Dd, or Td after which supernatants were collected and stored at −80°C (26). Unstimulated and pokeweed mitogen-stimulated cells served as negative and positive controls, respectively. The cytokines interferon-gamma (IFN-γ) (Th1), interleukin-13 (IL-13) (Th2), IL-17 (Th17), and IL-10 (Treg) were quantified in the supernatants using an in-house MIA developed according to de Jager et al. (27) and calibrated against the Bio-Plex cytokine assay kit (Bio-Rad Laboratories, Hercules, CA, USA).

### Statistical Analysis

Geometric mean concentrations with corresponding 95% confidence intervals were calculated for vaccine antigen-specific IgG, IgA, and cytokine concentrations. Numbers of vaccine antigen-specific memory B-cells are reported as geometric mean values/10^5^ CD19^+^ cells. The mean percent reduction of the IgG concentrations was calculated between the different time points after vaccination. The kinetics of IgG antibody levels was determined with a bi-exponential decay model as described (28).

Normal distribution of (log-transformed) data was confirmed prior to each analysis. Differences between time points were tested with paired sample *t*-tests (normal distributed data) or with Wilcoxon Signed Ranks tests (not normally distributed data). Correlations between variables were determined with Spearman correlations and linear regression analysis. A *p*-value <0.05 was considered statistically significant (two-sided test). Data were analyzed using GraphPad Prism v7 (GraphPad Software) and SPSS statistics v24 (IBM).

## Results

### Study Baseline Characteristics

**Figure 1:**
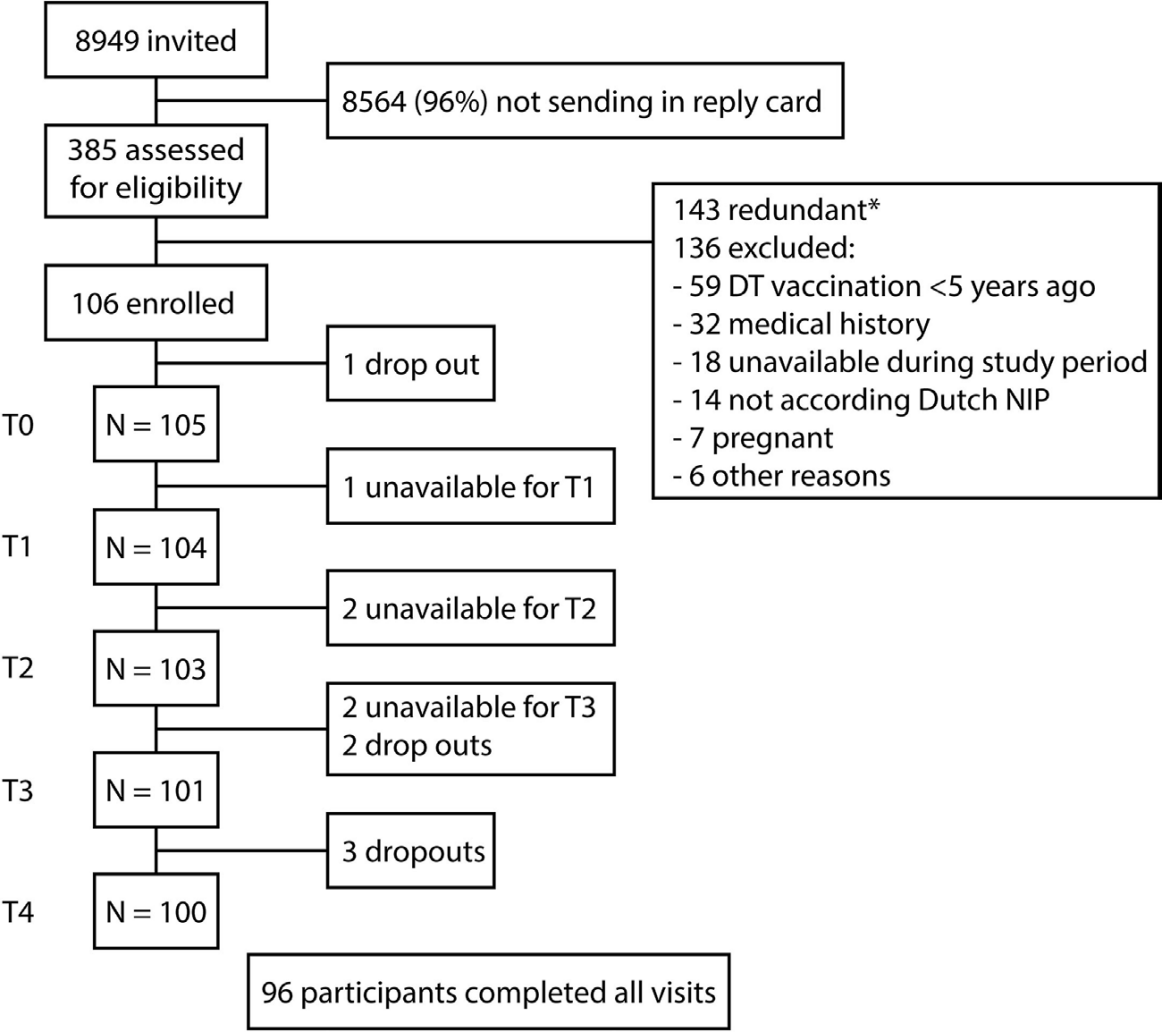
Flow-chart of study enrollment. Schematic overview of the recruitment, enrollment, and the follow-up of the study participants. At the start of the study, Dutch participants 25–29 years of age received a tetanus, diphtheria, and acellular pertussis booster vaccination. Blood samples were collected before (T0), 2 weeks (T1), 4 weeks (T2), 1 year (T3), and 2 years (T4) after the booster vaccination. *143 potential participants were excluded because the target for inclusion was achieved.

At the start of the study in April 2014, 105 participants received a Tdap booster vaccination (Figure 1). 2 years after the booster, 90.6% (96/106) of the participants completed all study visits and ≥100 blood samples had been collected at every time point. Mean age at the start of the study was 27.6 ± 1.4 years and 34% was male (36/106).

### IgG and IgA Antibody Kinetics After the Tdap Booster Vaccination

**Figure 2:**
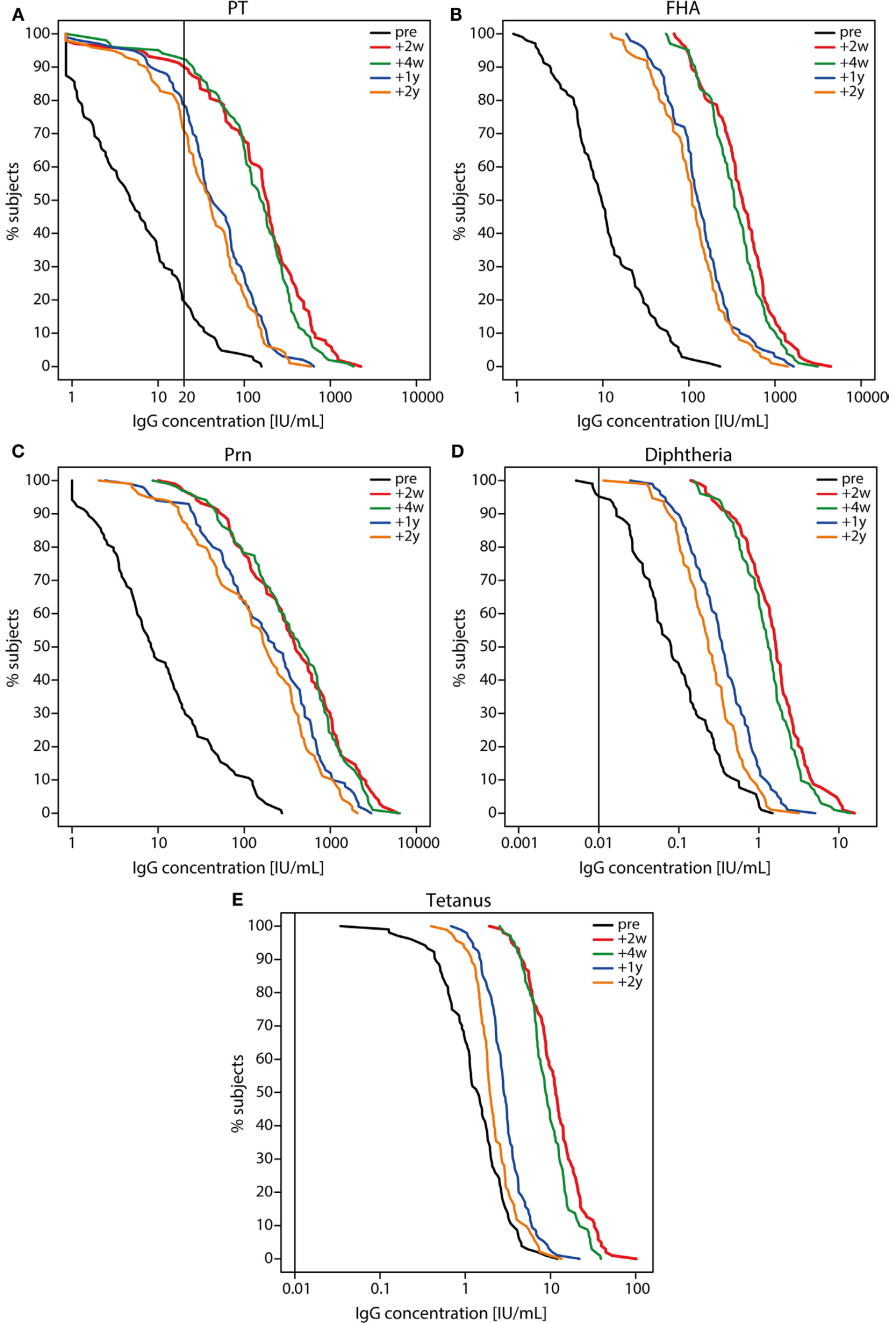
Reverse cumulative distribution curves of IgG levels before and after a tetanus, diphtheria, and acellular pertussis (Tdap) booster vaccination. **(A)** pertussis toxin (PT), **(B)** filamentous hemagglutinin, **(C)** pertactin, **(D)** diphtheria toxoid, and **(E)** tetanus toxin specific IgG levels (IU/mL) in Dutch adults 25–29 years of age before (pre; black lines), 2 weeks (red lines), 4 weeks (green lines), 1 year (blue lines), and 2 years (orange lines) after a first Tdap booster vaccination. Note, black line represents the cut-off for protection (PT 20 IU/mL (suggestive), diphtheria and tetanus 0.01 IU/mL).

Before the Tdap booster vaccination, IgG levels against PT, FHA, and Prn were low (Figure 2 and Table 1), although 7% (7/105) of the participants had a PT-IgG level ≥50 IU/mL. 1 and 2 years post-booster, this percentage increased to respectively 78% (79/101) and 71% (71/100) of the participants. Following the Tdap booster vaccination, the IgG levels increased for all vaccine antigens and remained higher at all time points compared with pre-booster levels (*p*-values <0.01) (Figure 2 and Table 1). Surprisingly, 8% (8/105) of the participants did not show a PT-IgG level ≥20 IU/mL at any of the time points. The IgG levels for diphtheria and tetanus were above the protective level pre-booster for most individuals and IgG levels increased upon the booster, but started to decline already after 2 weeks post-booster and progressed to decline significantly after 1 and 2 years post-booster (*p*-values <0.01) (Figure 2). The reduction in vaccine antigen-specific IgG varied between 41 and 66% in the first year, but was more limited (15–25%) during the second year post-booster (Table 1). Overall, the PT-IgG levels were similar between males and females, but 7/8 participants who did not have arbitrarily protective PT-IgG levels in the first month post-booster were females. At 2 years post-booster, more females showed PT-IgG levels under the protective cut-off (21/66 females, 32%) compared with males (8/34 males, 24%).

**Table 1:** IgG geometric mean concentrations (IU/mL) with 95% confidence intervals following a first adult tetanus, diphtheria, and acellular pertussis booster vaccination and percent reduction^a^ of IgG antibody levels.

	GMC IU/mL (95% CI)					Reduction (%)^a^	
		
	**Pre**	**+2 weeks**	**+4 weeks**	**+1 year**	**+2 years**	**4 weeks–1 year**	**1–2 years**
PT	5.4	130	123	43	35	58	15
	(4.1–7.2)	(95–178)	(93–161)	(33–55)	(27–45)		

FHA	10.6	404	339	132	108	53	17
	(8.4–13.2)	(340–480)	(288–399)	(110–158)	(90–131)		

Prn	10.5	360	357	180	142	41	20
	(7.8–14.0)	(270–481)	(269–473)	(132–247)	(104–194)		

Diphtheria	0.09	1.5	1.3	0.34	0.24	66	25
	(0.07–0.11)	(1.3–1.9)	(1.1–1.5)	(0.28–0.42)	(0.20–0.29)		

Tetanus	1.3	11.6	9.4	3.0	2.2	59	24
	(1.1–1.5)	(10.1–13.4)	(8.4–10.6)	(2.6–3.3)	(1.9–2.4)		

*GMC, geometric mean concentrations; CI, confidence intervals; PT, pertussis toxin; FHA, filamentous hemagglutinin; Prn, pertactin*.

*^a^Samples were excluded if no increase in antibody concentration was observed 2 weeks post-booster compared with pre-booster, or when an increase after 4 weeks post-booster was observed*.

**Figure 3:**
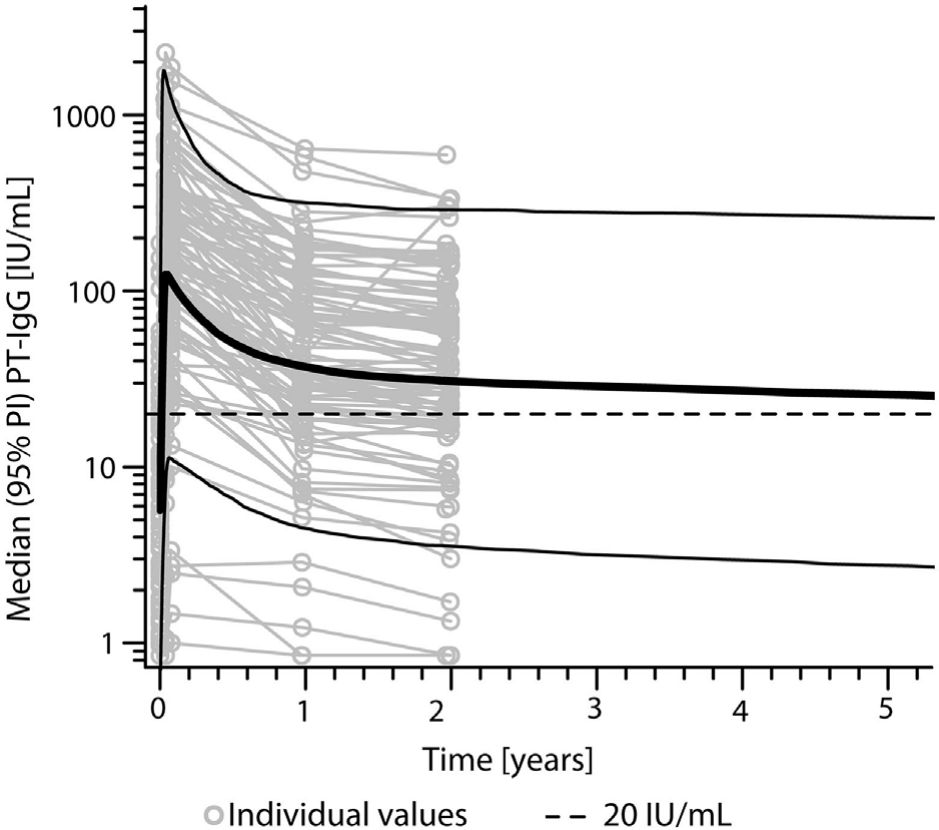
Predicted levels of pertussis toxin (PT)-specific IgG levels (IU/mL) after a first adult tetanus, diphtheria, and acellular pertussis booster vaccination in Dutch adults 25–29 years of age using a bi-exponential model. Note, solid bold line represents the median, solid lines represents the 95% predicted interval (95% PI), and dotted line represents a PT-IgG concentration of 20 IU/mL.

According to the bi-exponential model, the median PT-IgG level was predicted to remain above 20 IU/mL for approximately 9 years (Figure 3). Median duration of protection against diphtheria and tetanus both >0.01 IU/mL (29, 30) was predicted to approximately last 16 and 93 years, respectively post-booster.

**Figure 4:**
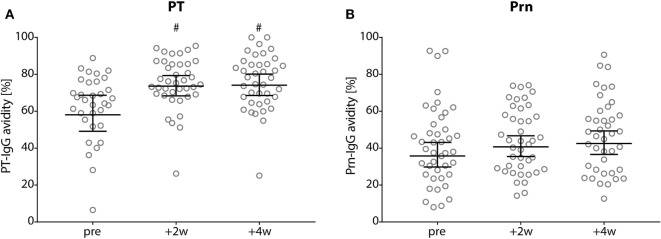
Avidity of pertussis toxin (PT)- and Prn-IgG antibodies. **(A)** PT and **(B)** pertactin avidity index in Dutch adults 25–29 years of age before (pre), 2 weeks, and 4 weeks after a first tetanus, diphtheria, and acellular pertussis booster vaccination. Note, black lines represents the geometric mean percentage with 95% confidence interval, ^#^*p*-value <0.01 compared with pre-booster levels.

The avidity (GMAI) of the PT-IgG antibodies was significantly higher for 2 and 4 weeks of post-booster compared with pre-booster (*p*-values <0.01), while no differences were observed in the GMAI of Prn-IgG antibodies (Figure 4).

**Figure 5:**
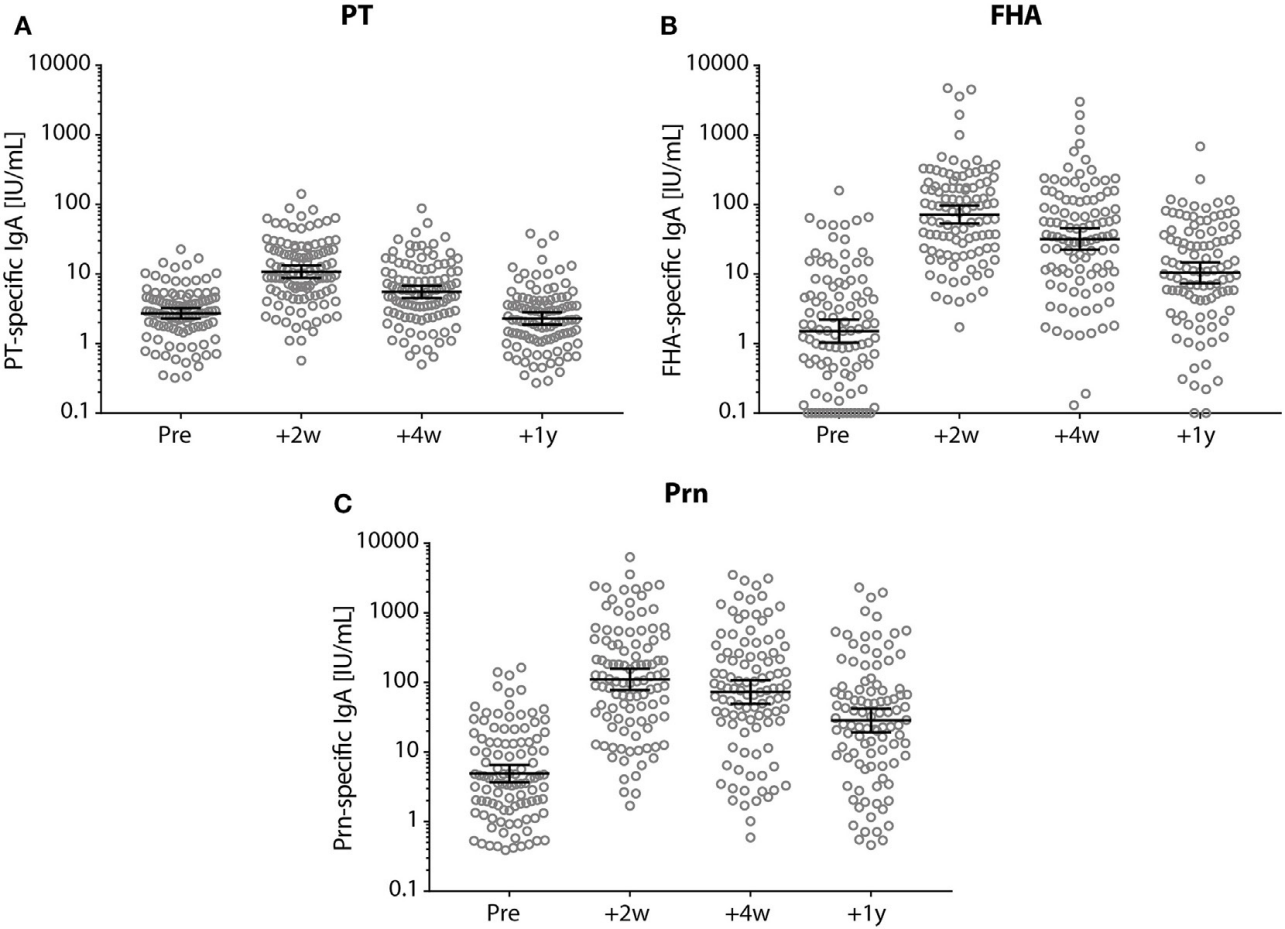
IgA antibody levels before and after a tetanus, diphtheria, and acellular pertussis (Tdap) booster vaccination. **(A)** Pertussis toxin (PT), **(B)** filamentous hemagglutinin, and **(C)** pertactin (Prn)-specific IgA levels (IU/mL) in Dutch adults 25–29 years of age before (pre), 2 weeks, 4 weeks, and 1 year after a first Tdap booster vaccination. Note, black lines represent the geometric mean concentration with 95% confidence interval. Each time point was significantly different compared with other time points.

Seropositive PT-IgA levels were observed in 85% (89/105) of the participants pre-booster, and in 99% (103/104) of the participants 2 weeks post-booster (Figure 5A). 2 and 4 weeks post-booster, the IgA levels for all three pertussis antigens had increased, but declined subsequently within the first year post-booster (*p*-values <0.001) (Figure 5).

### B-cell Responses After the Tdap Booster Vaccination

**Figure 6:**
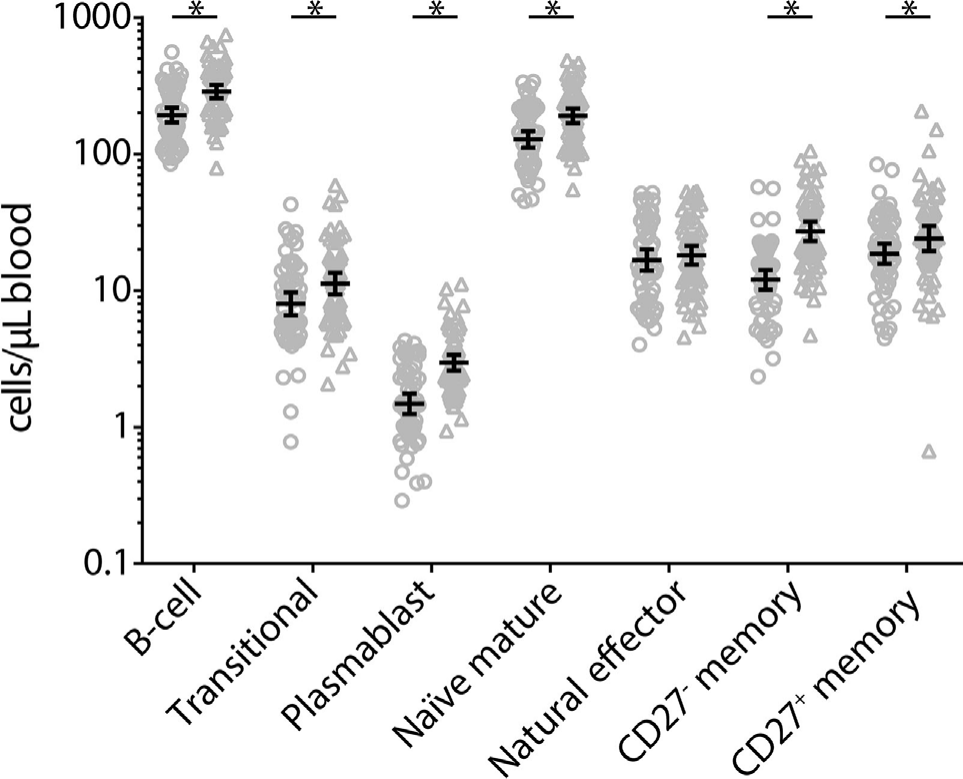
Absolute numbers of B-cell and B-cell subsets before (circles) and 2 weeks after (triangles) a first tetanus, diphtheria, and acellular pertussis booster vaccination in Dutch adults 25–29 years of age. Gating of populations (all SSC^low^): B-cell: CD45^+^CD19^+^; translational: CD45^+^CD27^−^CD38^+^; plasmablast: CD45^+^CD27^+^CD38^+^; naïve mature: CD45^+^CD27^−^IgD^+^CD38^dim^; natural effector: CD45^+^CD27^+^IgD^+^CD38^dim^; CD27^−^ memory: CD45^+^CD27^−^IgD^−^CD38^dim^; CD27^+^ memory: CD45^+^CD27^+^IgD^−^CD38^dim^. Note, black solid line represents the geometric mean numbers with corresponding 95% confidence intervals, *p-value <0.05.

**Figure 7:**
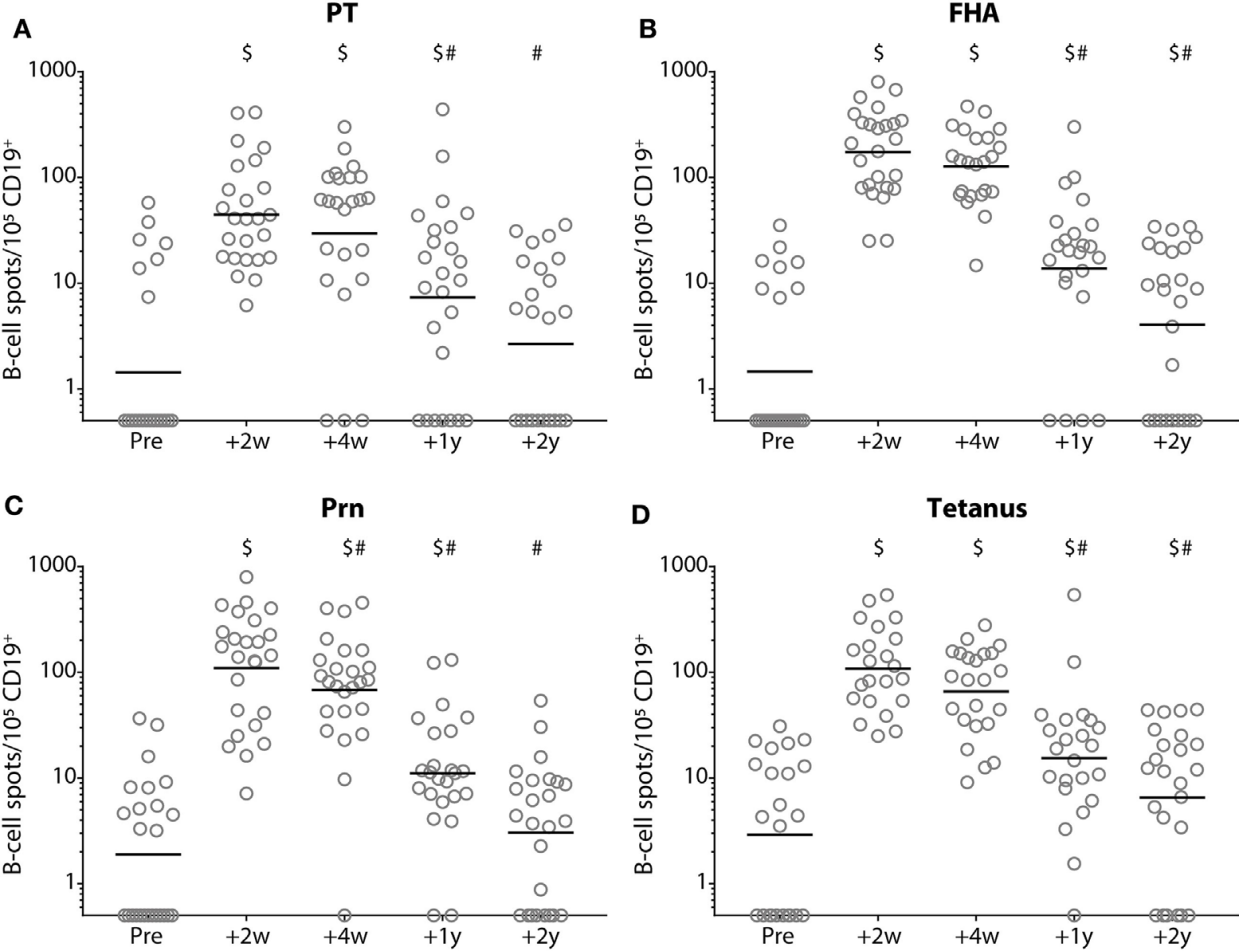
Numbers of memory B-cells before and after a tetanus, diphtheria, and acellular pertussis (Tdap) booster vaccination. The numbers of **(A)** pertussis toxin, **(B)** filamentous hemagglutinin, **(C)** pertactin, and **(D)** tetanus toxoid-specific IgG-producing memory B-cells/100,000 CD19^+^ cells in Dutch adults 25–29 years of age before (pre), 2 weeks, 4 weeks, 1 year, and 2 years after a first Tdap booster vaccination. Note, black solid line represents the geometric mean number, $ indicates significant increase compared with numbers pre-booster (*p*-value <0.05) and # indicates significant decrease compared with previous time point(s) post-booster (*p*-value <0.05).

**Figure 8:**
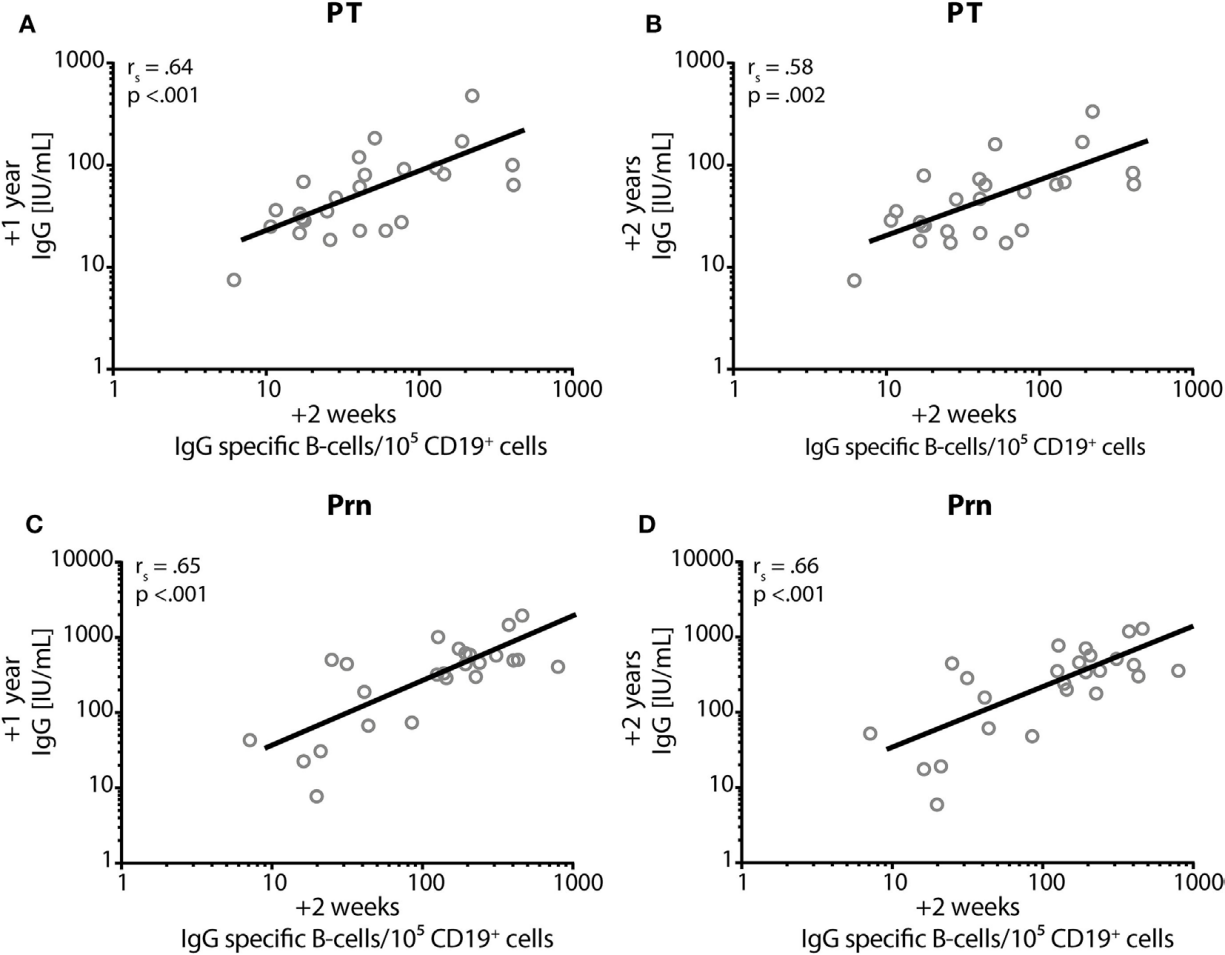
Correlation between IgG and memory B-cells. Spearman’s correlation coefficient (rs) between the numbers of IgG-specific memory B-cells/105 CD19^+^ cells (*x*-axis) at 2 weeks post-booster and the specific IgG levels in IU/mL (on the *y*-axis) at 1 year post-booster **(A,C)** and at 2 years post-booster **(B,D)** for pertussis toxin; **(A,B)** and for pertactin; **(C,D)**. *p* = *p*-value.

At 2 weeks post-booster, the absolute numbers of circulating B-cells and plasma-, naïve-, and memory-B-cell subsets had increased significantly compared with pre-booster numbers, except for natural effector B-cell numbers (Figure 6). Pre-booster, detectable numbers of vaccine antigen-specific circulating memory B-cells were observed in just a few participants (Figure 7). Following Tdap, the numbers of the vaccine antigen-specific memory B-cells/10^5^ CD19^+^ cells had increased significantly at all time points compared with pre-booster values, except for PT and Prn 2 years post-booster, but numbers had declined significantly between 4 weeks and 1 year post-booster (Figure 7). A correlation was observed between the numbers of specific memory B-cells at 2 weeks versus the specific IgG levels at 1 year and 2 years post-booster for PT and Prn (*r* = 0.64 and 0.58 for PT and *r* = 0.65 and 0.66 for Prn, respectively; *p*-values <0.01) (Figure 8).

### T-Cell Cytokine Responses After the Adult Tdap Booster Vaccination

**Figure 9:**
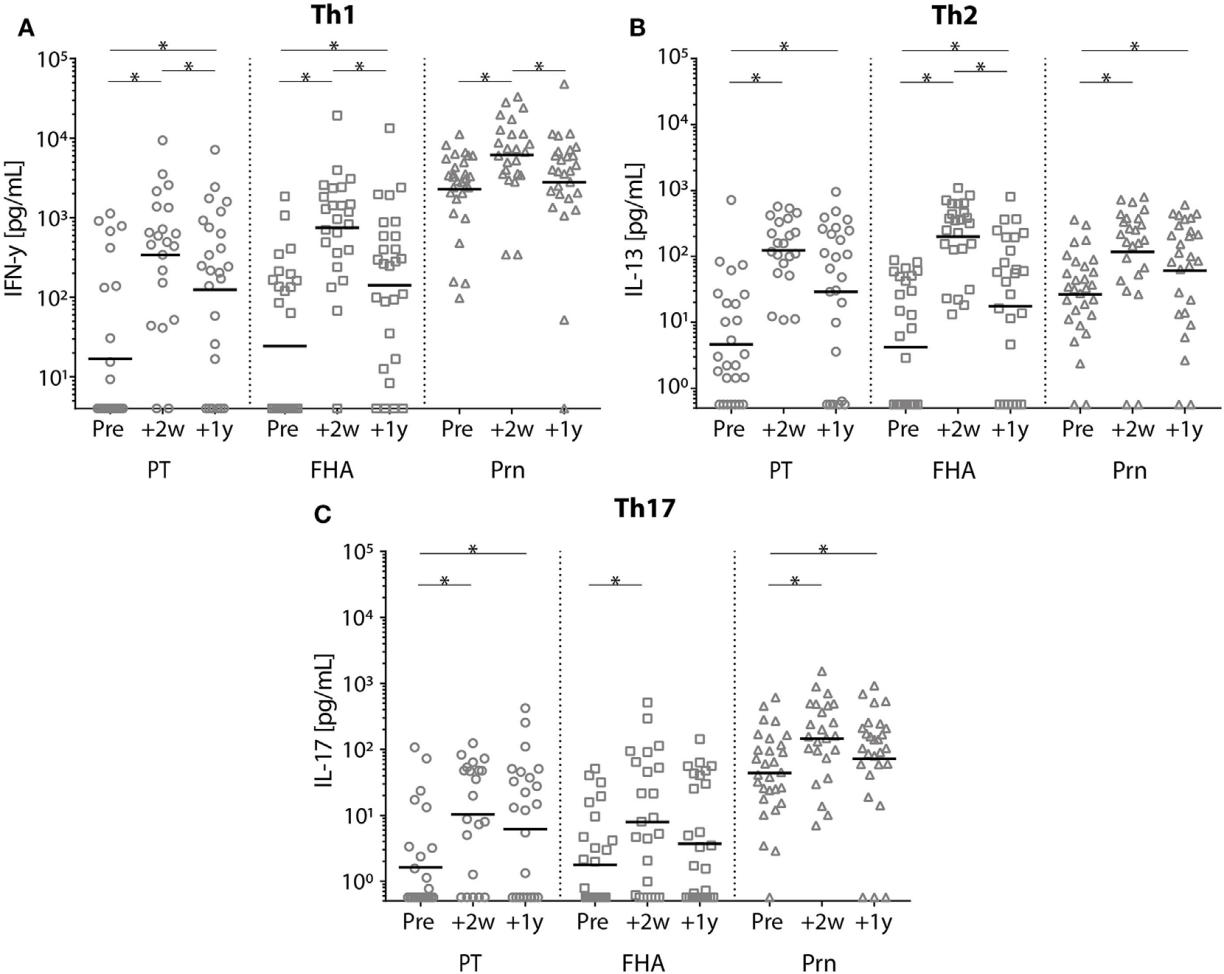
Cytokine levels of stimulated T-cells before and after a tetanus, diphtheria, and acellular pertussis (Tdap) booster vaccination. **(A)** Interferon-gamma, **(B)** interleukin-13, and **(C)** IL-17 cytokine concentrations (pg/mL) in the supernatants of T-cells stimulated with heat inactivated pertussis toxin (PT) (circles), filamentous hemagglutinin (squares), or pertactin (Prn) (triangles) in Dutch adults 25–29 years of age before 2 weeks and 1 year after a first Tdap booster vaccination. Note, black lines represents the geometric mean concentration; *p-value < 0.05.

In general, the production of IFN-γ, IL-13, and IL-17 cytokines increased significantly at all time points post-booster vaccination compared with pre-booster values (Figure 9; Figure S1 in Supplementary Material). Similar levels were observed at 2 and 4 weeks and again at 1 year and 2 years following Tdap (data 4 weeks and 2 years post-booster not shown). Pertussis-specific IFN-γ production and FHA-specific IL-13 production decreased significantly between 2 weeks and 1 year post-booster (Figure 9). Only low pertussis-specific IL-10 production was observed both pre- and post-booster (data not shown).

## Discussion

In this study, we demonstrated that systemic IgG levels against the pertussis vaccine antigens PT, FHA, and Prn persisted at higher levels for at least 2 years after a first adult aP booster vaccination in young Dutch adults 25–29 years of age, who had been primed in infancy with whole-cell pertussis vaccine. A limited antibody decay was observed during the second year post-booster with antibody levels against PT above 20 IU/mL in at least 70% of the participants and an estimated median duration of protection for about 9 years. The numbers of PT- and Prn-specific B-cells observed at 2 weeks post-booster correlated with the corresponding IgG antibody levels after 1 year and 2 years. Furthermore, the booster enhanced Th1, Th2, and Th17 cytokine production for at least 2 years.

Using the same vaccine antigen dose, the adult Tdap booster vaccination in this study induced 1.5–4 times higher PT-IgG levels compared with previous studies (15, 31, 32), which were conducted between 1997 and 2002 in a period with presumed lower circulation of *B. pertussis* than nowadays (17, 18). Our participants were, therefore, most likely more frequently exposed to *B. pertussis*. This is underlined by the fact that the majority of our participants showed pre-booster PT-IgA levels, that further increased post-booster. IgA responses are not induced by aP vaccines in infants (22), but exposure to *B. pertussis* induces systemic pertussis-specific IgA-producing memory B-cells in children and adults (22, 33). Therefore, the presence of IgA antibodies pre-booster and their rise post-booster may result from the activation of pre-existing pertussis-specific memory B-cells induced by previous *B. pertussis* contact in life, indicative of enhanced *B. pertussis* circulation nowadays. Together, the high pertussis circulation, compared to a population that is not or only minimally boosted with pertussis infections, alters pertussis immunity with enhanced antibody levels and cellular immunity upon a booster vaccination.

Using a bi-exponential antibody decay model with the arbitrarily defined level of protection of ≥20 IU/mL (23), the median duration of protective PT-IgG levels in our study was estimated to last up to 9 years after the Tdap booster. Although a power function decay model can also be used to determine antibody persistence, this model results in even more prolonged antibody persistence (34). Therefore, we prefer to use the more conservative bi-exponential model. We need to point out that the young adults in our study have been primed with wP vaccines during infancy, as is the case for the majority of the current adult Dutch population.

In this study, the highest pertussis-specific IgG levels were seen at day 14 post-booster followed by a slight decrease at day 28. In line with this, Halperin et al. reported increased IgG levels from day 7 with a peak around day 14 (35), and Kirkland et al. reported comparable IgG levels at 2 and 4 weeks, both after a Tdap booster (36), though using lower vaccine antigen concentrations compared with our study (35, 36). Other pertussis booster studies measured serological responses from 4 weeks post-booster onward (15, 37–39). Since peak IgG levels will be missed with sample collections from 4 weeks onward, vaccine antibody responses could be measured already around day 14 post-booster.

The higher IgG responses upon Tdap vaccination due to presumed recall of memory B-cells induced by a previous *B. pertussis* infection is reflected by the high numbers of pertussis-specific IgG memory B-cells after the booster, and the significantly higher absolute numbers of recirculating B-cell subsets. In line with this, Hendrikx et al. found similar numbers of PT-specific memory B-cells in pre-adolescents after a second aP booster vaccination. In contrast, post-booster numbers of FHA- and Prn-specific memory B-cells were higher in adults, probably a result of more natural boosting compared with pre-adolescents. The numbers of pertussis-specific circulating memory B-cells, a month after vaccination of adolescents correlate with antibody levels a year post vaccination suggesting that memory B-cells at least partly will differentiate into plasma cells upon vaccination (40). Although the homing of the pertussis-specific memory B-cells normally occurs quickly, waning circulating IgG levels during the second year was limited, probably by the presence of long-lived plasma cells in the bone-marrow. Others found no correlation between circulating memory B-cells and antibody levels for diphtheria and tetanus in steady state conditions. This suggests that peripheral memory B cells and antibody-secreting plasma cells may partly represent independently regulated cell populations and may play different roles in the maintenance of protective immunity (41). The induction of memory B-cells and long-lived plasma cells by the adult booster vaccination might contribute to long-term protection against pertussis.

In agreement with other studies, the adult booster vaccination resulted in increased levels of Th1, Th2, and Th17 cytokines (42, 43). However, Huygen et al. did not find increased Th1 levels upon the same vaccination in pregnant women or their age-matched controls (38). Also, a pre-adolescent Tdap booster in children 9 years of age did not enhance T-cell responses (44), which we explained at the time by the high pre-booster levels already induced by a booster vaccination 5 years earlier. So far, just one study investigated the influence of pertussis priming vaccines on adult T-cell responses after a Tdap booster vaccination (43). That study showed a general Th2-dominated immune response after an aP booster in adults primed with aP vaccines during infancy, while wP-primed adults showed a Th1 dominated response (43). Since Th1 cells are essential for bacterial clearance and associated with protection (45), the increased pertussis-specific Th1 levels observed in our wP-primed adults post-booster, could possibly confer protection against pertussis, while this may be less in aP-primed adults with a Th2-dominated response.

The increase in the number of pertussis-related deaths in infants during the epidemic of 2010 in California (10) and that of 2012 in the UK (11), led to the implementation of maternal pertussis booster vaccinations (9). Maternal aP vaccinations are very effective in preventing pertussis in infants in the time window from birth until their first routine pertussis immunization, even with low anti-PT antibody levels at 2 months of age (<15 IU/mL) (46–48). In several countries, pregnant women are advised to be vaccinated during every pregnancy (38, 49). The persistence of high PT-IgG antibody levels reported here could indicate that the repeated administration of a Tdap booster vaccination might not be necessary for the majority of pregnant women. IgG antibody kinetics after an aP booster vaccination in pregnant women should be studied in more detail. Also, attention must be paid to potential non-responders to aP vaccinations, since these comprise 10% (7/70) of our female study participants.

The switch from wP to aP vaccines during infancy in 2005, will bring the first Dutch aP-primed cohort reaching the age of 18 years in 2023. However, other countries have already used aP vaccines in the infant immunization program for more than two decades. Since protection against clinical pertussis wanes faster after priming with aP vaccines compared with wP vaccines (50), women at childbearing age primed with aP vaccines, may experience the consequences of less longer persistence of pertussis-specific antibodies after booster vaccination. Therefore, the effectiveness of adult and maternal Tdap vaccinations and antibody persistence deserves further study, accounting for previous vaccinations and current *B. pertussis* exposure in the population.

To conclude, we showed a robust immune response and persistence of high pertussis IgG antibody levels after a Tdap booster vaccination in Dutch adults 25–29 years of age. These adults have been primed with wP vaccines during infancy and might benefit from the booster vaccine by an elevated immune response to pertussis. Maternal aP vaccination is currently the best strategy to protect newborns from pertussis. Long-term follow-up of antibody levels in women vaccinated with aP during pregnancy could elucidate the necessity to vaccinate during every pregnancy. In addition, Tdap booster responses in growing cohorts of aP-primed individuals reaching childbearing age should be further investigated.

## Ethics Statement

Written informed consent was obtained at the start of the study. The study was approved by the Medical Ethics Review Committee North Holland (METC-NH, Alkmaar, the Netherlands) and registered at the European clinical trials database (2013-005355-32) and the Dutch trial register (www.trialregister.nl; NTR4494).

## Author Contributions

SL, GB, and A-MB were involved in the conception, planning, study design, and participant enrollment. SL, DR, MLB, MJB, and PG performed laboratory analysis. SL and AM performed statistical analysis. SL, ES, GB, and A-MB interpreted data and wrote the manuscript. All authors agreed to submit for publication.

## Conflict of Interest Statement

SL, DR, M-LZ-B, MB, PG, AM, GB, and A-MB have no conflicts of interest. ES declares to have received grant support for vaccine studies from Pfizer and GSK.
