# Supplementary material for: Robust Humoral and Cellular Immune Responses to Pertussis in Adults After a First Acellular Booster Vaccination
Source: Front Immunol. 2018 Apr 4;9:681. doi: 10.3389/fimmu.2018.00681 (PMC5893963; doi:10.3389/fimmu.2018.00681)
Supplement: Supplementary file 1 [file image_1.PDF]

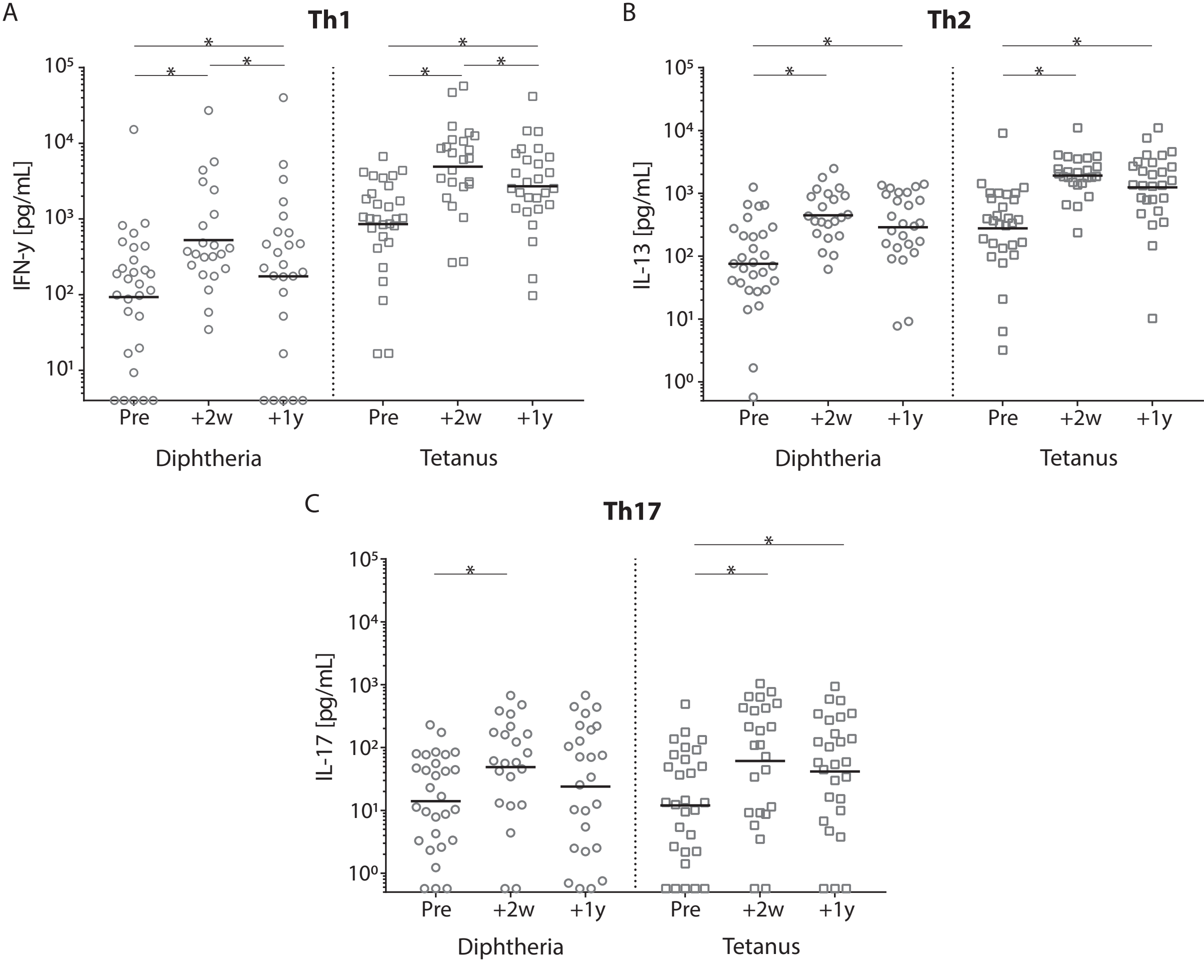

Supplementary Figure 1. Cytokine levels of stimulated T-cells before and after a Tdap booster vaccination. (A) IFN-γ, **(B)** IL-13, and **(C)** IL-17 cytokine concentrations (pg/mL) in the supernatants of T-cells stimulated with diphtheria toxoid (circles) or tetanus toxoid (squares) in Dutch adults 25 to 29 years of age before, 2 weeks and 1 year after a first Tdap booster vaccination. Note, black lines represents the geometric mean concentration; * = p-value <.05.
